# Gut Microbiome Dysbiosis in Alcoholism: Consequences for Health and Recovery

**DOI:** 10.3389/fcimb.2022.840164

**Published:** 2022-03-03

**Authors:** Andrew Whittier Day, Carol A. Kumamoto

**Affiliations:** ^1^ Graduate School of Biomedical Sciences, Tufts University, Boston, MA, United States; ^2^ Department of Molecular Biology and Microbiology, Tufts University, Boston, MA, United States

**Keywords:** microbiome, mycobiome, alcoholism, alcoholic liver disease, gut-brain axis

## Abstract

Since the mid 1980’s, the impact of gastrointestinal (GI) microbiome changes during alcohol use disorder has been an area of significant interest. This work has resulted in the identification of specific changes in the abundance of certain members of the GI microbiome and the role these changes play in a variety of alcohol related disorders (*i.e.* alcoholic liver disease). Interestingly, some findings suggest a possible role for the GI microbiome in alcohol addiction or withdrawal. Unfortunately, there is a significant gap in knowledge in this area. Here we describe differences in the GI microbiome of alcoholic and non-alcoholic individuals and discuss the possible impact of microbes on the gut-brain axis, which could impact alcohol related behaviors (*i.e.* addiction). Understanding the role of the GI microbiome in alcohol related disorders will potentially lead to the development of successful microbiome-targeted therapeutics to help mitigate these disorders.

## Introduction

Alcoholism contributes significantly to global morbidity and mortality as roughly 5.3% of annual deaths result from harmful consumption of alcohol (WHO, 2018). In younger age groups, alcohol abuse poses an even greater threat with 13.5% of all deaths of people from ages 20-39 attributed to alcohol use and abuse (WHO, 2018). Importantly, the COVID-19 pandemic has significantly contributed to higher relapse rates of alcoholics, a rise in alcoholic liver disease (ALD), and alcoholic deaths (Knopf, 2021; Sarangi and Eskander, 2021). The general decline in mental health, increased rates of addiction in comparison to previous decades, and the rapid escalation during the pandemic warrants a greater understanding of addiction and mental health pathogenesis.

Alcoholism has been studied for many years, with most of the research focusing on its impacts on the brain. A less studied, important area of recent research has been the examination of gastrointestinal (GI) microbiome changes in alcoholic patients. The first observation was made by Bode et al., who reported that jejunal aspirates from alcoholic individuals more commonly contained gram-negative anaerobic bacteria compared to control individuals (Bode et al., 1984). Mutlu and co-workers reported that more than ten weeks of ethanol ingestion in rats led to significant dysbiosis of the colonic microbiome suggesting that the composition of the microbiota may be important in ALD (Mutlu et al., 2009). In subsequent years, many sequencing studies of the microbiome from rodent models of alcoholism, humans with alcohol use disorder (AUD), as well as non-human primate studies of addiction have been conducted.

More recently, several studies have begun elucidating the possible roles of the microbiota in ALD (Kakiyama et al., 2014; Bajaj et al., 2017; Yang et al., 2017; Chu et al., 2020; Lang et al., 2020b; LeBrun et al., 2020). These studies highlight a role for bacterial and fungal microbiota in the progression of ALD; however, the effects of dysbiosis on other organ systems such as the brain are less understood. Work by Leclercq and co-workers showed that microbiome alterations in alcoholism and decreased intestinal barrier integrity can have profound effects on the central nervous system leading to increases in depression, anxiety, and alcohol craving (Leclercq et al., 2014). Furthermore, in a subsequent study, Leclercq et al. showed that humans with AUD have changes in serum metabolites from the kynurenine/tryptophan pathway compared to control individuals. Production of neuroprotective kynurenic acid (KYNA) is decreased in humans with AUD and production is shifted to yield an increase in the neurotoxic metabolite quinolinic acid (QUIN), resulting in a decreased ratio of KYNA/QUIN. Moreover, the ratio of KYNA/QUIN positively correlates with fecal abundance of the genus *Faecalibacterium*. Plasma levels of tryptophan and KYNA were also shown to negatively correlate with depression and alcohol craving, respectively (Leclercq et al., 2021).

These studies highlight the possibility that the intestinal dysbiosis observed in alcoholics could perpetuate and promote addiction through alterations to metabolism and neuronal pathways. Throughout this review, we will compile findings from studies performed on human GI microbiome samples from alcoholic individuals and highlight patterns of changes seen in these individuals. Moreover, we will examine the work characterizing the involvement of the microbiome in ALD and the brain. Finally, we will discuss promising microbiome-directed therapeutics for the treatment of AUD.

## Bacterial Microbiome Changes in the Alcoholic Gastrointestinal Tract

Many of the early studies of the alcoholic microbiome focused on establishing the presence of dysbiosis in the GI tract of alcoholics and a possible association with ALD (Mutlu et al., 2009; Chen et al., 2011; Mutlu et al., 2012). These studies noted phylum level changes in the alcoholic GI tract, such as increased Firmicutes and decreased Bacteroidetes (Chen et al., 2011; Mutlu et al., 2012; Tsuruya et al., 2016; Addolorato et al., 2020; Smirnova et al., 2020). However, these types of changes may not be specific to alcoholism as they are also associated with many other conditions such as obesity, consumption of a high-fat diet (Ley et al., 2005; Hildebrandt et al., 2009; Koliada et al., 2017), aging (Mariat et al., 2009), poor cardiovascular health (Yang et al., 2015), and treatment of gastroesophageal reflux disease (Clooney et al., 2016).

**Figure 1:**
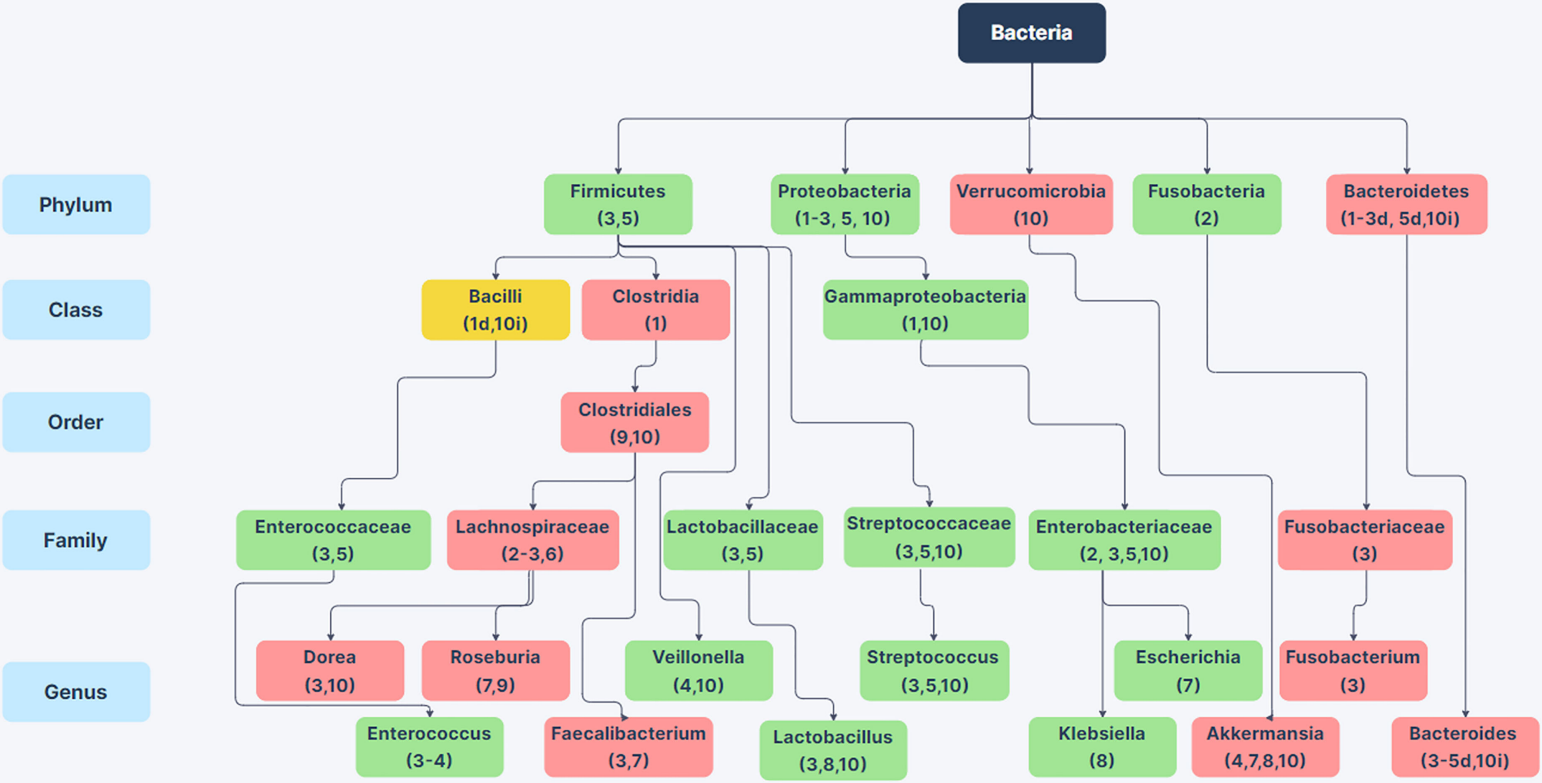
Changes in Bacterial Abundance in the Alcoholic GI Tract. Selected phylum, class, order, family, and genus-level differences in bacterial abundance between the gut microbiome in patients with alcohol-use disorder or varying stages of alcoholic liver disease compared to non-alcoholic humans from 10 human studies. Green boxes indicate taxa with increased abundance in alcoholics (in at least 75% of studies) and red boxes indicate taxa with decreased abundance in alcoholics (in at least 75% of studies). Yellow boxes indicate conflicting information between studies. Citations are noted below the bacterial name. For taxa with conflicting information, d or i next to the citation indicates decreased or increased abundance in the alcoholic GI tract. References: (1) Mutlu et al., 2012; (2) Chen et al., 2011; (3) Smirnova et al., 2020; (4) ; Lang et al., 2020b; (5) Tsuruya et al., 2016; (6) Ames et al., 2020; (7) Gurwara et al., 2020; (8) Dubinkina et al., 2017; (9) Seo et al., 2020; (10) Addolorato et al., 2020. Created using Zen Flowchart (www.zenflowchart.com).

Sub-phylum level comparisons between the intestinal microbiota of non-alcoholic humans and individuals with AUD or varying stages of ALD have provided a clearer picture of alcoholism related changes in GI microbiota. **Figure 1** shows a compilation of 10 studies examining the microbiome of alcoholic humans and shows the variability between sub-taxa of a phylum in terms of whether the taxa increase or decrease in alcoholics. It is worth mentioning that some of these studies compare patients with various stages of ALD to control populations that do not abuse alcohol, so some of the changes in these specific studies could be related to liver dysfunction rather than substance abuse. At the phylum level, Firmicutes are notable as these organisms comprise the majority of the microbiome in humans and numerous members of the Firmicutes phylum show changes in abundance between the microbiota of non-alcoholic and alcoholic individuals. Within the Firmicutes phylum, there are noted changes at the genus level such as decreases in anti-inflammatory *Faecalibacterium* (Leclercq et al., 2021), decreases in *Roseburia* which has implications for gastrointestinal barrier integrity (Seo et al., 2020), increases in genera containing opportunistic pathogens such as *Streptococcus* and *Enterococcus*, and increases in *Lactobacillus* (Lang et al., 2020b; Smirnova et al., 2020; Tsuruya et al., 2016; Dubinkina et al., 2017; Addolorato et al., 2020). Additionally, there are increases in the Proteobacteria phylum and the class Gammaproteobacteria in alcoholics (Mutlu et al., 2012; Addolorato et al., 2020); many members of this class are enteric pathogens. To our knowledge, it is not known whether alcoholic individuals have more infections originating from the GI tract. This is an important gap in our knowledge that warrants future study.

Family level changes that are seen in alcoholic microbiota include increases in Enterococcaceae, Lactobacillaceae, Streptococcaceae, and Enterobacteriaceae; decreases in Lachnospiraceae and Fusobacteriaceae (**Figure 1**) (Chen et al., 2011; Tsuruya et al., 2016; Addolorato et al., 2020; Ames et al., 2020; Smirnova et al., 2020). Genus-level changes that are seen are largely decreases in obligate anaerobic bacteria such as *Bacteroides, Akkermansia, Roseburia, Faecalibacterium*, and in some studies *Bifidobacterium* (**Figure 1**) (Tsuruya et al., 2016; Dubinkina et al., 2017; Addolorato et al., 2020; Gurwara et al., 2020; Lang et al., 2020b; Seo et al., 2020; Smirnova et al., 2020). Genera that increase in the alcoholic GI tract are largely facultative anaerobes such as *Enterococcus*, *Escherichia, Klebsiella, Lactobacillus*, and *Streptococcus* (Lang et al., 2020b; Smirnova et al., 2020; Tsuruya et al., 2016; Dubinkina et al., 2017; Addolorato et al., 2020; Gurwara et al., 2020). The enrichment of facultative anaerobes in the alcoholic GI tract suggests the possibility that facultative anaerobes could originate from aerobic areas of the GI tract such as the oral cavity and stomach. Although there is sparse data on oral microbiome changes in AUD, one study showed that the oral microbiome is enriched for *Lactobacillus, Prevotella, Streptococcus, and Veillonella*, all of which are enriched in alcoholic fecal samples (Ames et al., 2020). The results from these studies show that the alcoholic gut microbiota is dysbiotic, with reductions in butyrate producing bacteria such as Clostridia (Mutlu et al., 2012), reductions of beneficial bacteria such as *Akkermansia, Roseburia, Faecalibacterium and Bacteroides*, and overrepresentation of Gammaproteobacteria, other pathobionts, and many facultative anaerobes as shown in **Figure 1**.

These changes lead to many questions about how the environment of the alcoholic GI tract differs from that of non-alcoholic individuals and what consequences these changes have to the host. There may be a greater abundance of reactive oxygen species (ROS) in the alcoholic GI tract due to repeated consumption of ethanol and constant metabolism of the alcohol; ethanol metabolism generates ROS through various routes including Cytochrome P450-dependent mechanisms (Wu and Cederbaum, 2003; Cederbaum, 2012; Tsuruya et al., 2016). The changes in bacterial microbiome composition in the alcoholic GI tract may partly be due to ROS-tolerance (Tsuruya et al., 2016). However, more work is needed to decipher the environment within the alcoholic gut, how it impacts the microbiome, and how alterations in amounts of specific intestinal microbes affect different tissues.

## Fungal Microbiome Changes in the Alcoholic Gastrointestinal Tract

While bacteria make up most of the GI microbiome, roughly 10^11^ bacteria per gram of feces (Sender et al., 2016), there are other important members of the microbiome such as archaea, fungi, and viruses. Fungi make up a smaller portion of the fecal microbiota, about 10^6^ fungal cells per gram of feces, but represent an important factor in interactions of the microbiome with the host, specifically during fungal blooms following bacterial dysbiosis due to antibiotic use (Rosenbach et al., 2010; Wang et al., 2014; Yang et al., 2017). Fungi have also been shown to increase in abundance in the GI tract following diets rich in meats and cheeses (David et al., 2014), recent carbohydrate consumption (Hoffmann et al., 2013), and most notably for this review: chronic alcohol consumption (Yang et al., 2017). Fungi represent a reservoir of opportunistic pathogens that cause bloodstream infections with high mortality rates. For example, bloodstream infections by *Candida* species have mortality rates of 56% in individuals with cirrhosis (Bartoletti et al., 2014). Within the last few years, the fungal microbiome, or mycobiome, has been a topic of interest in individuals with AUD and various stages of ALD.

**Figure 2:**
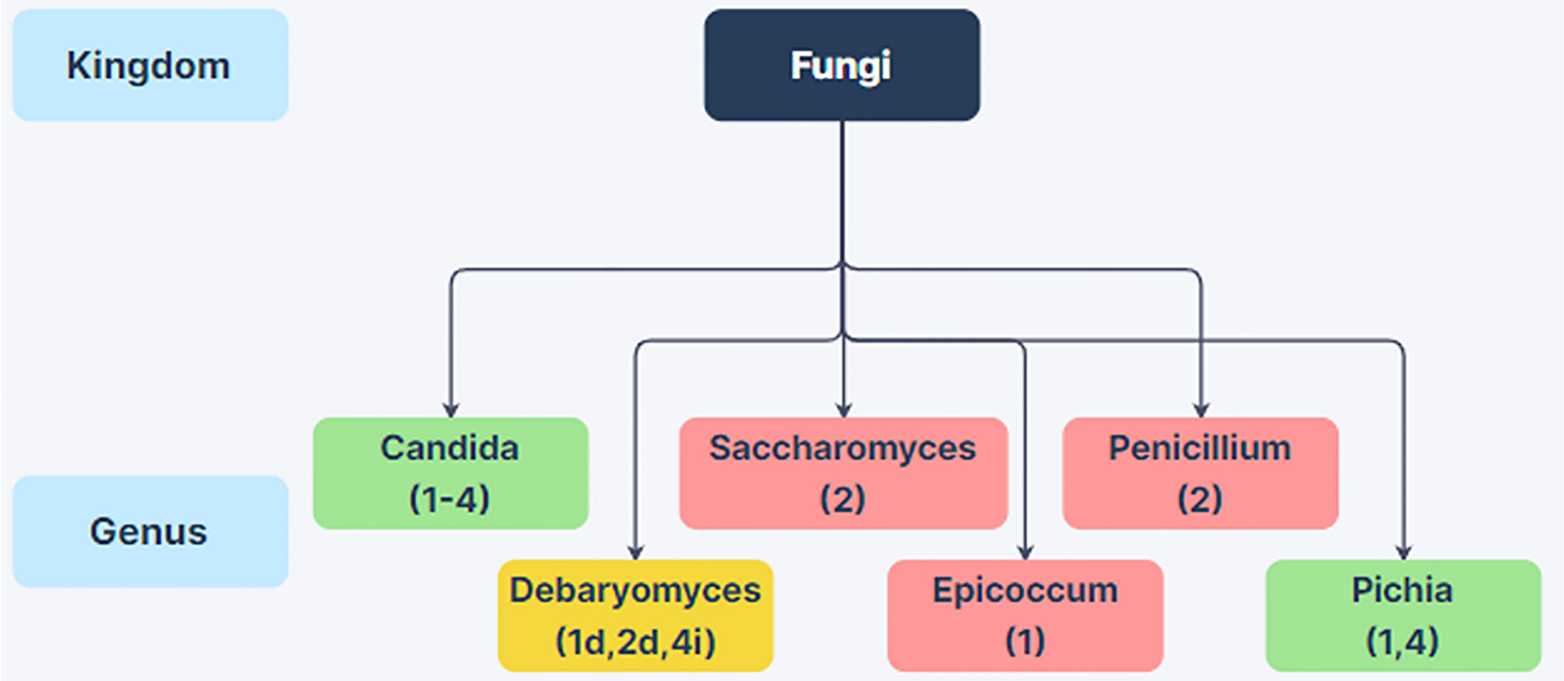
Changes in Fungal Abundance in the Alcoholic GI Tract. Genus-level differences in fungal abundance between the gut mycobiome in patients with alcohol-use disorder or varying stages of alcoholic liver disease relative to non-alcoholic humans from 4 human studies. Green boxes indicate increased abundance in alcoholics and red boxes indicate decreased abundance in alcoholics. Yellow boxes indicate conflicting information between studies. Citations are noted below the fungal name. Taxa with conflicting information have decreased or increased abundance in the alcoholic GI tract noted with a d or i next to the citation, respectively. References: (1) Yang et al., 2017; (2) Lang et al., 2020a; (3) Chu et al., 2020; (4) Hartmann et al., 2021. Created using Zen Flowchart (www.zenflowchart.com).

While there have been many bacterial microbiome studies of alcoholics elucidating phylum through genus level changes spanning the last 15 years, there have only been a few studies investigating mycobiome composition changes. Many of these studies have only examined genus level changes, and thus the mycobiome changes in the alcoholic GI tract are not well understood. However, there are interesting differences in the mycobiome of alcoholics compared to non-alcoholic individuals **(** **Figure 2** **)**. Each study has noted an increase in the abundance of *Candida* species, and two have reported an increase in the genus *Pichia* (**Figure 2**). *Saccharomyces, Penicillium, Epicoccum* have all been reported to decrease in the alcoholic GI tract (Yang et al., 2017; Chu et al., 2020; Hartmann et al., 2021; Lang et al., 2020a). Conflicting reports have been obtained for *Debaryomyces* (Yang et al., 2017; Hartmann et al., 2021; Lang et al., 2020a). These reports raise the possibility that alcoholics could be at greater risk of infection from pathobionts such as *Candida* spp. and *Pichia* spp. Individuals with increased *Debaryomyces* could have a reduced ability to heal alcohol-related damage to the intestinal barrier as *Debaryomyces hansenii* has been shown to inhibit wound healing (Jain et al., 2021). However, more work is needed to examine possible impacts of intestinal mycobiome changes in these regards.

The mechanisms leading to fungal dysbiosis in the alcoholic GI tract remain unclear. One possibility is that fungi are taking advantage of disruption to normal bacterial homeostasis as fungal blooms have been reported following bacterial dysbiosis (Rosenbach et al., 2010; Wang et al., 2014; Yang et al., 2017). However, further research is needed to examine why fungal genera such as *Candida* and *Pichia* increase in abundance in the alcoholic GI tract.

## The Gut-Brain Axis in Alcoholism

Alcoholism and other addictions are extremely complex disorders. Throughout addiction development, individuals acquire a multitude of cognitive changes and dysfunctions characterized by alterations to emotional processing, memory, executive functioning, and goal-oriented behavior (Le Berre et al., 2017). Along with these changes, it has been known for decades that functional changes to the GI tract can occur in alcoholics. Most notably GI changes can result in nutritional deficiencies in alcoholics including deficits in vitamin A (possible role in liver disease) (Lieber, 2003), thiamine [role in Wernicke’s encephalopathy and Korsakoff’s syndrome (WKS)] (Coulbault et al., 2021), and niacin (pellagra and WKS) (Narasimha et al., 2019; Yoshii et al., 2019; Lewis, 2020). Before severe physical complications such as WKS, pellagra, or end-stage liver disease occur, there are years of addiction that represent a critical time window for intervention. In this window, many alcoholic individuals suffer from psychiatric comorbidities such as depression, anxiety, bipolar disorder, schizophrenia, and PTSD (Chambers, 2013; Bradizza et al., 2018). These conditions perpetuate addiction and make recovery more difficult (Chambers, 2013; Bradizza et al., 2018). This complex picture painted by addiction could be affected by GI populations of bacteria and fungi through the gut-brain axis (GBA).

The GBA is defined as the bidirectional communication of the brain and GI tract, and many recent reports have shown the role that gut microbes can have on the GBA (reviewed in Stilling et al., 2014). Different pathways for communication between the gut and the brain include neuronal signaling through the vagus nerve, endocrine effects through the hypothalamic-pituitary-adrenal axis, stimulation of neural inflammation, or a wide range of changes to metabolism (reviewed in Bonaz et al., 2018). Many studies have shown that bacteria impact the gut-brain axis and are implicated in conditions and phenotypes such as autism spectrum disorders, social behavior, anxiety, depression, food preference, and food consumption amount (Bravo et al., 2011; Vijay-Kumar et al., 2010; Duca et al., 2012; Dash et al., 2015; Mayer et al., 2015; Vuong and Hsiao, 2017; Ntranos and Casaccia, 2018; Jang et al., 2018a; Jang et al., 2018b; Shan et al., 2020).

One noted phenotype that can impact the gut-brain axis in some of these different disorders is increased intestinal permeability (Mayer et al., 2015). Increased intestinal permeability is described as an intestinal epithelium that loses barrier integrity (sometimes termed “leaky gut”). Increased intestinal permeability can result in systemic release of microbial products leading to inflammation and potentially to depression (Dash et al., 2015).

*Escherichia coli* has been shown to lead to inflammation and cause anxiety-like phenotypes in mice through NF-κB-dependent pathways and hippocampus involvement (Shan et al., 2020). *Klebsiella oxytoca* colonization in mice leads to a similar outcome with an increased anxiety-like phenotype following increased inflammation through NF-κB-dependent pathways (Jang et al., 2018b). Moreover, microbial alterations to metabolism, most notably changes in abundance of short-chain fatty acids (SCFAs) such as acetate, propionate, and butyrate, have been shown to affect many disorders such as depression, anxiety, stress, autism, and schizophrenia (Dalile et al., 2019; Taylor and Holscher, 2020).

Bacteria are not the only microbes known to impact the GBA. Colonization by the commensal fungus, *Candida albicans* has also been shown to cause an anxiety-like phenotype in mice through alterations to lipid metabolism and the endocannabinoid pathway (Markey et al., 2020). Many genera implicated in GBA disorders have a higher abundance in the GI tract of alcoholics (*Klebsiella, Escherichia*, and *Candida*). The potential roles of these taxa in substance use disorders are not fully elucidated.

Some bacteria have protective effects. For example, *Lactobacillus rhamnosus* improved anxiety-like and depression-related phenotypes through the vagus nerve (Bravo et al., 2011). *Bifidobacterium* has also been shown to play a protective role, as it improved depression-related phenotypes in mice through alterations to 5-HT (serotonin) metabolism (Tian et al., 2019).

While there have been many studies describing microbial impacts on the GBA, the microbiota-gut-brain axis in alcoholism has been relatively unexplored. Recently there have been interesting and noteworthy studies describing how the alcoholic microbiome could affect the brain in AUD. A recent study showed that giving mice a fecal microbiota transfer from alcoholic humans led to altered behavior, specifically reduced social behavior, through reductions of β-hydroxybutyrate, and increased depression in mice that received the transplant (Leclercq et al., 2020). One of the first studies to examine the role that the microbiome could play in promoting alcoholism was done in 2014 (Leclercq et al., 2014). In this study, alcohol-dependent patients with higher intestinal permeability had significantly higher levels of withdrawal-associated depression and anxiety than alcohol-dependent patients with low intestinal permeability. The level of intestinal permeability also significantly correlated with the intensity of depression, anxiety, and craving during withdrawal. Furthermore, there were marked changes in the intestinal microbiomes between the high and low intestinal permeability groups. Intestinal permeability showed significant negative correlation with the abundance of the protective organism *Bifidobacterium* spp. (Riedel et al., 2006) and the anti-inflammatory bacterium *Faecalibacterium prausnitzii* (Miquel et al., 2015). Reductions in protective species could increase intestinal permeability and psychiatric disorders that negatively impact alcoholism. In fact, subsequent work showed that serum metabolites in the tryptophan/kynurenine pathway correlated with depression, anxiety, and craving (Leclercq et al., 2021). The concentrations of kynurenine and neurotoxic quinolinic acid negatively correlated with the relative abundance of *Faecalibacterium* in the GI tract, and abundance of *Faecalibacterium* positively correlated with the ratio of neuroprotective kynurenic acid to quinolinic acid— suggesting a role for this bacterial genus in metabolism of neuroprotective kynurenic acid (Leclercq et al., 2021). These studies highlight a likely involvement of the microbiome in alcoholism-associated pathologies through impact on the GBA. There are likely many more metabolic changes contributing to GBA dysfunction in the alcoholic GI tract as the gut microbiome plays vital roles in metabolism of SCFAs, amino acids, and other carbon sources (Oliphant and Allen-Vercoe, 2019)

*Faecalibacterium prausnitzii* is not the only bacterium hypothesized to have a protective role in the alcoholic GI tract. Seo et al. described a role for *Roseburia* spp. in protecting intestinal barrier integrity in the alcoholic GI tract and maintaining functional glycan metabolism (Seo et al., 2020). Flagellin from *Roseburia* increased the intestinal barrier integrity in an alcoholic mouse model. Further, levels of *Roseburia* were significantly reduced in alcoholic patients compared to their non-alcoholic twin. Additional experimental study of *Roseburia*, *Faecalibacterium* and their effects on the gut-brain axis are needed to elucidate roles for these bacteria in alcoholism.

Identification of new microbes that impact the GBA in alcoholism is needed to gain a greater understanding of microbial impact on the brain. While there have been few studies examining the effects the microbiome has on the GBA in alcoholism, there have been many studies explaining the impact the GI microbiome has on ALD. The liver represents another player in the GBA in AUD, with its known involvement in neurological diseases and conditions such as hepatic encephalopathy and neuroinflammation (Butterworth, 2013). There are far more studies examining the impacts of the microbiome on ALD and thus should be considered when examining connections of the microbiome and the GBA.

## Impact of Bacterial and Fungal Microbiome Changes on Alcoholic Liver Disease

Effects of the microbiome on ALD have been the topic of many investigations. There are many noted examples of microbes impacting the health and function of the liver such as hepatitis viruses and their contributions to hepatocellular carcinoma (Arzumanyan et al., 2013), aflatoxin ingestion produced by *Aspergillus* species and its role in liver cancers (Marchese et al., 2018), and many important interactions between the microbiome and the liver that are central to bile acid production and other metabolic reactions (Wahlström et al., 2016). Microbial interactions with the liver are quite common as the liver represents one of the first environments that microbes and microbial products encounter following intestinal epithelium leakage (Tilg et al., 2016). As noted above, there is a marked decrease in intestinal barrier integrity in individuals with AUD (Varella Morandi Junqueira-Franco et al., 2006; Keshavarzian et al., 2009; Swanson et al., 2015) and therefore in the alcoholic GI tract, there is likely extensive interaction of the liver with bacteria, fungi, and microbial products. Studying these interactions is key to our understanding of later stages of disease progression.

Many studies identify effects of microbiota on ALD. In 2016, Llopis, et al. studied the composition of the fecal microbiome of patients with alcoholic hepatitis. To test for functional effects of the microbiome, they transplanted microbiota from humans with or without alcoholic hepatitis into germ free mice and observed significant increases in liver weight, leukocyte infiltration, and T-cell infiltration in the livers of the mice that received microbiota from humans with alcoholic hepatitis (Llopis et al., 2016). When these recipient mice were given access to alcohol, there were significant increases in liver damage markers in the mice receiving the microbiota from humans with alcoholic hepatitis. This study thus showed the importance of the microbiota in promoting ALD.

Several studies have focused on studying specific microbial players such as *Enterococcus faecalis* (Llorente et al., 2017; Duan et al., 2019) that drive ALD progression. Llorente et al. showed that taking proton pump inhibitors (PPIs) led to a suppression of gastric bile acid secretion and resulted in a higher susceptibility of alcoholic individuals to the development of ALD. Patients taking PPIs had increased intestinal populations of *E. faecalis* that led to higher numbers of culturable *E. faecalis* in the liver and increased hepatic inflammation to promote ALD in an IL-1β-dependent manner (Llorente et al., 2017).

Additionally, other studies indicate a role for commensal fungi in ALD progression. Specifically, Yang et al. have shown that there is fungal dysbiosis in fecal samples of patients with ALD (Yang et al., 2017). They showed that the mycobiome of patients with alcoholic hepatitis or alcoholic cirrhosis were comprised almost completely of two fungal genera: *Candida* and *Pichia*. They further showed that patient survival correlated with the serum titer of anti-fungal IgG such that patients with higher titers had a significant decrease in 5-year survival (Yang et al., 2017). They proposed a model by which products of the fungal cell wall, β-glucan, accumulate in the alcoholic liver and drive inflammation through Kupffer cell activation and increases in IL-1β. In a subsequent study, Chu et al. showed that fecal levels of *Candida albicans* were higher in patients with alcoholic hepatitis. *C. albicans* exotoxin, or Candidalysin, damages hepatocytes in a dose-dependent manner in cell culture and increases liver inflammation to increase the severity of liver disease in alcoholic mouse models (Chu et al., 2020). Thus, there appear to be key bacterial taxa, fungal taxa, and microbial products involved in the progression and severity of ALD.

## Possible Microbiome Directed Therapies

With the noted involvement of the gut microbiome in addiction-related psychological disorders and ALD, it is possible that the alcoholic microbiome could be targeted to improve recovery outcomes in patients with AUD and cirrhosis. In fact, microbiome-directed therapies have been the topic of prior investigation by several labs such as bacteriophage treatment of cytolysin-positive *E. faecalis* to improve ALD outcomes (Duan et al., 2019), *Bifidobacterium* and *Lactobacillus* probiotic treatment for ALD (Kirpich et al., 2008; Stadlbauer et al., 2008), and fecal microbiota transplantation (FMT) for the improvement of treatment outcomes in patients with ALD (Philips et al., 2017). More recently, FMT has been examined as a possible treatment aid in early recovery. Bajaj et al. explored the use of FMT for the treatment of AUD in a phase 1 clinical trial (Bajaj et al., 2021). The group enrolled 20 alcoholic men to address safety and impact of FMT for the treatment of AUD and saw that FMT resulted in a reduction of serum IL-6, reductions in craving, cognitive functioning improvements, and reduction in negative psychosocial impacts (Bajaj et al., 2021). The strategy this group used addressed a lower abundance of intestinal populations of *Lachnospiraceae* and *Ruminococcaceae* by selecting an FMT-donor with higher abundance of these families of bacteria. Notably, two genera in these families have been implicated to have a protective role on the GBA and intestinal epithelium in alcoholism: *Faecalibacterium* and *Roseburia* (Leclercq et al., 2014; Seo et al., 2020; Leclercq et al., 2021). The group noted an increased abundance of *Roseburia* in FMT-recipients specifically (Bajaj et al., 2021). This study highlights the possibility that restoration of beneficial bacteria could improve central nervous system health in early recovery. FMT could thus represent another important tool that could be used in the battle against alcoholism. With more studies highlighting important bacteria and fungi that impact the GBA of alcoholics, FMT strategies could be targeted further to improve treatment efficacy.

## Discussion

There are clear changes to specific bacterial and fungal taxa in the alcoholic GI tract. The studies examined in this review identified consistent changes in the abundance of several taxa in the GI tract. With comparisons between individuals with different stages of ALD, from different areas around the world, and consuming different diets, it was expected that some taxa would show inconsistent, potentially stochastic, differences in abundance. Given these limitations, it is likely that many of the consistent changes in abundance illustrated in **Figures 1** and **2** are related to high consumption of alcohol in AUD. Therefore, genera with consistent differences in abundance between alcoholics and non-alcoholics should be considered for future study for a role in AUD-related pathologies and phenotypes. Additionally, future studies should address potential effects on microbiota and AUD due to host factors such as ethnicity and race, to determine the best future individualized microbiome-directed therapies.

**Figure 3:**
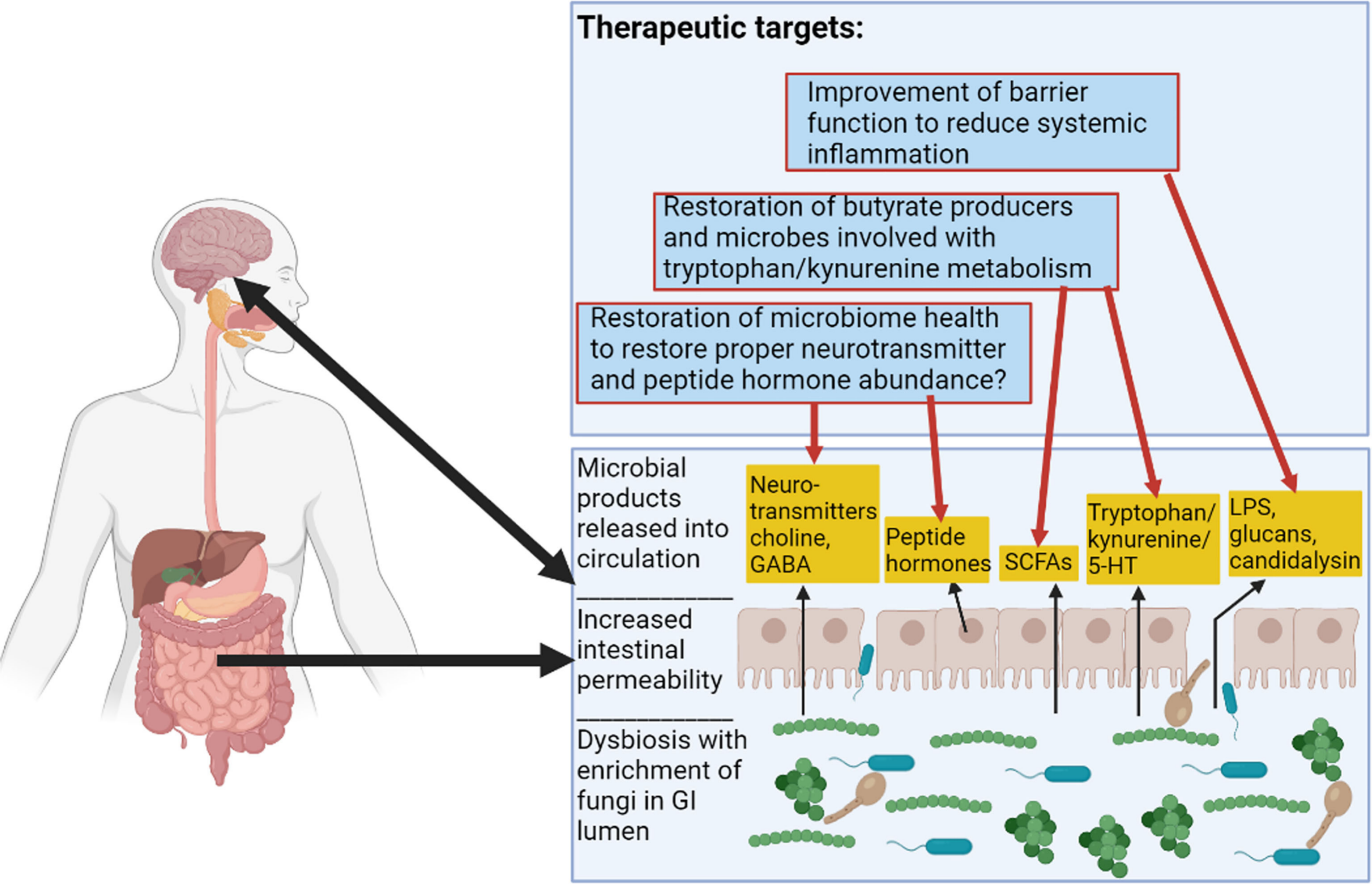
Alcoholic Microbiome-Related Therapeutic Targets for Future Interventions. This figure illustrates alterations to the GI tract in alcoholism. There are functional changes such as alterations to SCFA (short-chain fatty acid) levels, alterations to tryptophan/kynurenine and 5-HT (serotonin) metabolite levels, increased intestinal permeability resulting in proximal and distal inflammation, and alterations to levels of hormones and neurotransmitters. Therapeutic targets are described in the top box and red arrows show some previously considered targets and speculative therapeutic targets pertaining to the microbiome in alcoholism. Figure created using BioRender.com.

Future therapies could include strategies such as improving GI epithelial barrier integrity, increasing intestinal populations of butyrate producing bacteria, or altering kynurenine and tryptophan metabolism. These goals have been addressed by the FMT strategy used by Bajaj et al., 2021, either directly or indirectly. These possible FMT-based and probiotic strategies are illustrated in **Figure 3**. Other potential targets for microbiome therapeutic strategies include gastrointestinal hormones implicated in alcoholism such as ghrelin, leptin, and GLP-1, as well as neurotransmitters implicated in the alcoholic GBA, such as serotonin and dopamine (**Figure 3**) (Mittal et al., 2017; Farokhnia et al., 2019). More work is needed to elucidate microbiome strategies that could restore altered concentrations of these molecules in patients with AUD.

Much of the research to date has examined possible roles these changes have on pathogenesis of ALD. However, there have been noted impacts that the alcoholic microbiome as a whole or certain members have on the GBA. Certain genera with observed changes in the alcoholic GI tract have been implicated in affective disorders such as anxiety and depression. Therefore, additional studies on genera such as *Candida*, *Klebsiella, Escherichia*, and others will contribute to a more comprehensive understanding of the GBA in AUD. Further, the constant exposure of microbes to ethanol, its biproducts, and other metabolites in the alcoholic GI tract could drive microbial evolution and select for microbial populations occupying new metabolic niches or altering their interactions with the host. All of these factors are important to consider when studying this complex system to gain a better understanding and select the most efficacious therapeutics. Further research to study the GBA, identify new microbes involved, and extend the promising results obtained with initial FMT studies in alcoholism represent a collective effort to target the microbiome in AUD. Addressing these different areas of research can better target future microbiome directed therapies and improve treatments for AUD.

## Author Contributions

Conceptualization: AD and CK. Preparation of the first draft: AD. Editing of the manuscript: AD and CK.

## Funding

AD was supported by training grant T32AI007422 from the National Institutes of Health (to R. Isberg). Research in the Kumamoto lab was supported by grant R01 AI118898 from the National Institutes of Health (to CK).

## Conflict of Interest

The authors declare that the research was conducted in the absence of any commercial or financial relationships that could be construed as a potential conflict of interest.

## Publisher’s Note

All claims expressed in this article are solely those of the authors and do not necessarily represent those of their affiliated organizations, or those of the publisher, the editors and the reviewers. Any product that may be evaluated in this article, or claim that may be made by its manufacturer, is not guaranteed or endorsed by the publisher.
